# OntoMaton: a Bioportal powered ontology widget for Google Spreadsheets

**DOI:** 10.1093/bioinformatics/bts718

**Published:** 2012-12-24

**Authors:** Eamonn Maguire, Alejandra González-Beltrán, Patricia L. Whetzel, Susanna-Assunta Sansone, Philippe Rocca-Serra

**Affiliations:** ^1^Oxford e-Research Centre, University of Oxford, 7 Keble Road, OX1 3QG, Oxford, UK and ^2^Stanford Center for Biomedical Informatics Research, Stanford University, 1265 Welch Road, CA 94305, USA

## Abstract

**Motivation:** Data collection in spreadsheets is ubiquitous, but current solutions lack support for collaborative semantic annotation that would promote shared and interdisciplinary annotation practices, supporting geographically distributed players.

**Results:** OntoMaton is an open source solution that brings ontology lookup and tagging capabilities into a cloud-based collaborative editing environment, harnessing Google Spreadsheets and the NCBO Web services. It is a general purpose, format-agnostic tool that may serve as a component of the ISA software suite. OntoMaton can also be used to assist the ontology development process.

**Availability:** OntoMaton is freely available from Google widgets under the CPAL open source license; documentation and examples at: https://github.com/ISA-tools/OntoMaton.

**Contact:** isatools@googlegroups.com

## 1 INTRODUCTION

Well-annotated and shared bioscience research data offer new discovery opportunities and drive science of the future. Several data management plans and sharing policies have emerged, along with a growing number of community-developed guidelines and ontologies to harmonize the reporting of experiments from different domains so that these can be comprehensible and in turn, reproducible and reusable. In many research projects however, the generation and collection of experimental data occur in a multicentric, distributed fashion; and a variety of data types are generated often in a single experiment. Use of spreadsheets and related editors, such as Microsoft Excel for collecting experimental description is widespread among researchers due to their flexibility, low learning curve and above all ubiquity of tooling. However, misalignment, conflicting versions, the heterogeneity of free text, and also silent and unwanted ‘auto-corrections’ are major shortcomings to be addressed (Zeeberg *et al.*, 2004). This scenario and the current budgetary restrictions require ‘invest to save’ solutions to promote consistent annotation and collaborative editing of bioscience experiments, assisting researchers in complying with reporting policies and community standards. OntoMaton is an open source tool that leverages the collaborative environment and editing functionalities brought by Google Spreadsheets, and provides access to ontology look-up and tagging functionalities served by the NCBO BioPortal and Annotator web services (Jonquet *et al.*, 2010; Whetzel *et al.*, 2011).

## 2 OntoMaton DESIGN AND USE CASES

Four main use cases drove the development of OntoMaton: (i) to allow collaborative, distributed and coordinated annotation while enabling configurations and restrictions to be defined; (ii) to reduce free text description in metadata tracking of experimental data; (iii) to assist design patterns-based ontology development by facilitating interaction with domain experts; and (iv) to ease mapping between models and semantic representations. Two use cases are discussed more specifically in the next sections: one insisting on the free form of the widget and its ability to integrate in any layout, agnostic of any framework; the other aligning with a standardization effort, the ISA syntax (Sansone *et al.*, 2012). While ontology-enabled standalone tools exist (Rocca-Serra *et al.*, 2011; Wolstencroft *et al.*, 2011), they lack collaborative features. OntoMaton, with the aid of the Google Spreadsheet environment delivers this. OntoMaton is implemented in JavaScript upon the Google App Script API and accesses the NCBO RESTful web services. A webcast tutorial of how to use it is available at http://goo.gl/FjghA.

## 3 COLLABORATIVE SEMANTIC ANNOTATION

**Fig. 1.**
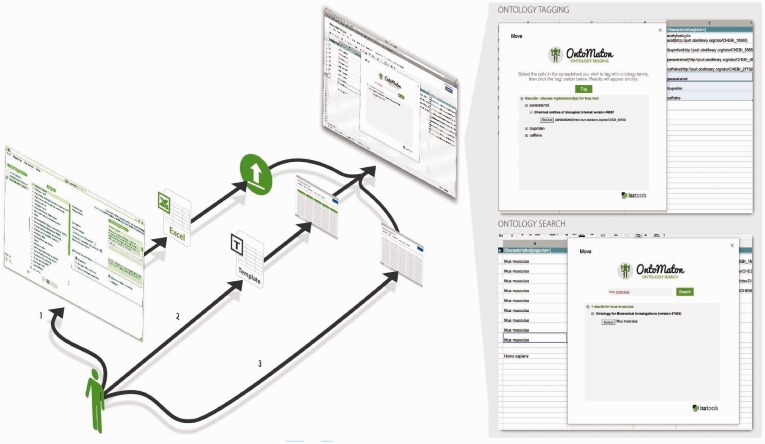
Uses of OntoMaton: (1) open the OntoMaton-enabled Google Spreadsheet template from the online gallery; (2) create a standard Google Spreadsheet and install OntoMaton within that; or (3) as part of the ISA suite, export an Excel template from ISAconfigurator and upload it into Google Spreadsheets

The OntoMaton Google widget can be installed and invoked from any Google Spreadsheet document or embedded in Google Templates. It provides a facility for searching ontologies hosted at NCBO BioPortal, or for calling a tagging functionality, by relying on NCBO’s Annotator services (Jonquet *et al.*, 2010). An OntoMaton-enabled Google spreadsheet can also be configured to restrict the ontological search space to specific resources. While OntoMaton is syntax neutral, its usefulness is demonstrated when exploited by a data management infrastructure, for instance to support the creation of ISA-Tab compatible templates. The ultimate goal is to foster adoption of reporting standard conformant spreadsheets for managing biological experimental data description. Figure 1 provides detailed alternative uses of OntoMaton and a snippet of an experiment being marked-up. Several data management projects—with an existing large user base are currently using OntoMaton-based templates to assist with their data collection and management needs. These include: the Earth Microbiome Project (http://goo.gl/JLG5d); Bioplatforms Australia (http://goo.gl/uXLve)—with a focus on soil metagenomics sample collection; and Metabolights (Steinbeck *et al.*, 2012)—a repository of metabolite profiling data at the European Bioinformatics Institute.

## 4 COLLABORATIVE ONTOLOGY ENGINEERING

Developing ontologies and knowledge representation artefacts requires the interaction of domain experts and computer scientists. The core interaction consists of converting domain expert vetted representations (a.k.a a design pattern) to OWL representations through the intervention of knowledge engineers. Tools such as Populous (Jupp *et al.*, 2012) and Protege Mapping Master (http://protege.stanford.edu) have been developed to support these activities. The developers of the Ontology of Biomedical Investigations (OBI) (Brinkman *et al.*, 2010), currently rely on the Quick Term Template (Rocca-Serra *et al.*, 2011) approach to quickly add defined classes based on a template and the Manchester OWL Syntax for the mapping. However, owing to the collaborative nature of OBI development, the approach has been hindered by the lack of tools. OntoMaton closes this gap and several templates have now been documented to support different design patterns. Those templates unfold the restrictions of a class model in a table: fields correspond to facet fillers and cell values should be class names or URIs. OntoMaton, by enabling *in situ* resource lookups, simplifies development, review and curation by the pool of OBI editors.

## 5 DISCUSSION

Developed by harnessing the Google Spreadsheet environment and the term lookup and annotation power of the NCBO Web services, OntoMaton is an effective tool assisting both collaborative semantic annotation of experiments and the ontology development process. Several annotation tools exist (Nelson *et al.*, 2011) but not all have support for community-driven guidelines and ontologies, and none of them allow collaborative annotation. Moreover, Excel-based tools (Jones and Côté, 2008) tend to be platform and version dependent. Google Spreadsheets on the other hand work across all platforms. A comparison of tools attempting to mix spreadsheets with access to vocabulary servers is available at http://goo.gl/NV3lZ. Ongoing development of OntoMaton focuses on: (i) transformation of data into the Resource Description Framework and Linked Data; (ii) support for cell level, vocabulary drop-down list as soon as the Google API supports it; and (iii) further integration with the ISA software suite as requested by users.

*Funding*: This work was supported by the Biotechnology and Biological Sciences Research Council [grant BB/I025840/1, BB/I000771/1 and BB/I000917/1 to S.A.S.] and the National Institutes of Health [grant U54 HG004028 supporting TW].

*Conflict of Interest*: none declared.
